# 290. Persistence of Long COVID in SARS-CoV-2 Confirmed Cases One-Year Post Infection

**DOI:** 10.1093/ofid/ofab466.492

**Published:** 2021-12-04

**Authors:** Harrison L Howe, Danielle A Rankin, Sean M Bloos, Kailee N Fernandez, Seifein Salib, Rana Talj, Danya Waqfi, Jessica Villarreal, Ahmad Yanis, James Chappell, Leigh Howard, Natasha B Halasa, Natasha B Halasa

**Affiliations:** 1 Vanderbilt University Medical Center, Goodlettsville, Tennessee; 2 Vanderbilt University Medical Center; Division of Pediatric Infectious Diseases, Nashville, TN

## Abstract

**Background:**

Regardless of severity of acute SARS-CoV-2 illness, adults infected with SARS-CoV-2 are at risk for post-acute sequelae of COVID-19. Long COVID is typically classified as symptoms lasting greater than four weeks post-infection. We aimed to evaluate the frequency of resolved and unresolved long COVID symptoms in adults residing in greater Nashville, TN.

**Methods:**

We conducted a longitudinal cohort study of SARS-CoV-2-positive and exposed individuals from March 20 to May 15, 2020. Participants for this analysis were included if: 1) ≥18 years; 2) SARS-CoV-2 positive by molecular or antibody testing; and 3) completed a one-year visit. Demographic and illness information were collected at enrollment, and long COVID symptoms were systematically collected at the one-year survey. Long COVID symptoms are defined as an adult experiencing at least one of the following symptoms four weeks post-infection: fatigue, confusion, loss of smell or taste, shortness of breath, chest pain, cough, muscle aches, inability to exercise, or heart palpitations. Unresolved symptoms are defined as an individual with long COVID still experiencing symptoms at the one-year visit.

**Results:**

A total of 115 adults enrolled and completed the one-year survey, of which 63 (54.8%) were SARS-CoV-2-positive, with one asymptomatic individual. Of SARS-CoV-2-positive symptomatic adults, 32 (51%) were female, 5 (88%) were of Hispanic ethnicity, and 58 (92%) were white. At the one-year visit, 33 (52%) reported having long COVID, of which 17 (52%) reported having unresolved symptoms. Fatigue (89%), headache (89%), muscle aches (79%), and cough (77%) were the most common symptoms reported at illness onset (Figure 1). Among 33 adults with long COVID, fatigue (42%), loss of smell (39%), and loss of taste (33%) were most common (Figure 2A). In the 17 individuals with unresolved symptoms, loss of smell (29%) and loss of taste (24%) were commonly reported (Figure 2B).

Figure 1. COVID-19 symptoms reported at enrollment (n=62)

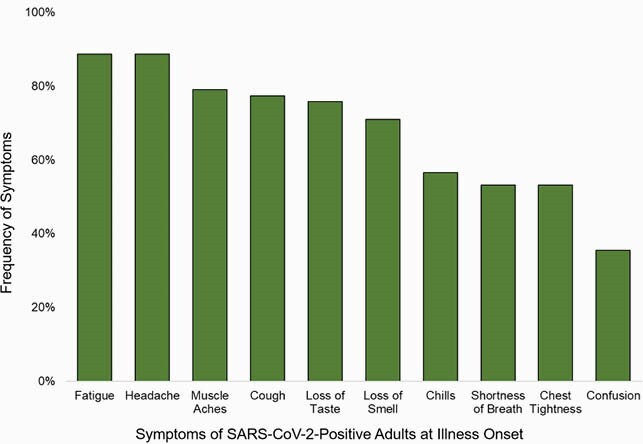

Figure 2. Long COVID (symptoms lasting ≥ 4 weeks) (n=33) (A) and unresolved long COVID symptoms one-year post-infection (n=17) (B) reported on the one-year survey

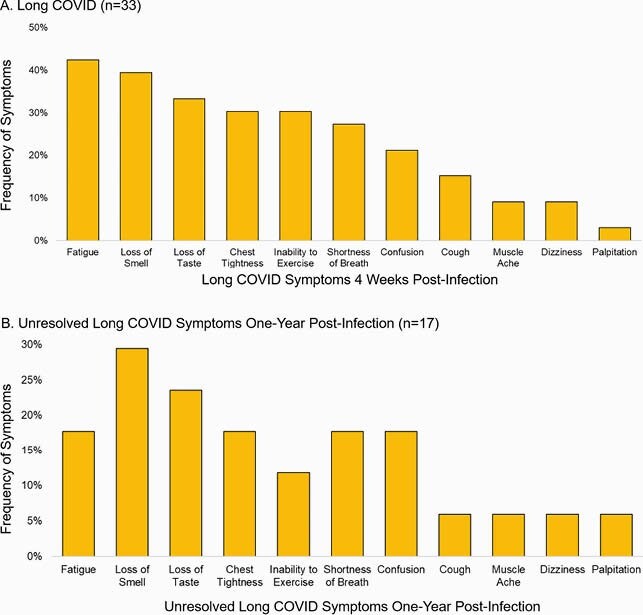

**Conclusion:**

Half of the adults in our cohort reported long COVID symptoms, with more than quarter of symptoms persisting one-year post-illness. Our findings support that prolonged symptoms up to year after SARS-CoV-2 exposure occur, and future studies should investigate the residual impacts of long COVID symptoms and conditions.

**Disclosures:**

**Natasha B. Halasa, MD, MPH**, **Genentech** (Other Financial or Material Support, I receive an honorarium for lectures - it’s a education grant, supported by genetech)**Quidel** (Grant/Research Support, Other Financial or Material Support, Donation of supplies/kits)**Sanofi** (Grant/Research Support, Other Financial or Material Support, HAI/NAI testing) **Natasha B. Halasa, MD, MPH**, Genentech (Individual(s) Involved: Self): I receive an honorarium for lectures - it’s a education grant, supported by genetech, Other Financial or Material Support, Other Financial or Material Support; Sanofi (Individual(s) Involved: Self): Grant/Research Support, Research Grant or Support

